# Kinetic disruption of lipid rafts is a mechanosensor for phospholipase D

**DOI:** 10.1038/ncomms13873

**Published:** 2016-12-15

**Authors:** E. Nicholas Petersen, Hae-Won Chung, Arman Nayebosadri, Scott B. Hansen

**Affiliations:** 1 Departments of Molecular Therapeutics and Neuroscience, The Scripps Research Institute, Scripps, Florida 33458, USA

## Abstract

The sensing of physical force, mechanosensation, underlies two of five human senses—touch and hearing. How transduction of force in a membrane occurs remains unclear. We asked if a biological membrane could employ kinetic energy to transduce a signal absent tension. Here we show that lipid rafts are dynamic compartments that inactivate the signalling enzyme phospholipase D2 (PLD2) by sequestering the enzyme from its substrate. Mechanical disruption of the lipid rafts activates PLD2 by mixing the enzyme with its substrate to produce the signalling lipid phosphatidic acid (PA). We calculate a latency time of <650 μs for PLD activation by mixing. Our results establish a fast, non-tension mechanism for mechanotransduction where disruption of ordered lipids initiates a mechanosensitive signal for cell growth through mechanical mixing.

## Abstract (web summary)

Mechanosensation by biological membranes can be relayed by mechanical tension to ion channels. Here the authors show that phospholipase D (PLD) is activated by mechanical disruption of lipid rafts which allows PLD to mix with its substrate in the lipid membrane, and propose a kinetic model of force transduction.

Mechanotransduction is the process by which mechanical force is converted into a chemical or electrical signal. It is the underlying mechanism for both touch and hearing and is known to have roles in cancer, allodynia heart and vascular disease^1^,^2^. The plasma membrane is thought to couple force directly with effector molecules such as mechanosensitive ion channels^3^,^4^,^5^ and organize mechanosensitive proteins including focal adhesion proteins^6^. These mechanosensitive proteins often reside compartmentalized within or outside of lipid rafts^7^.

Classically, work (*W*) done on an object increases the energy of that object through the application of force (*W*=*Fd*). If an object is fixed, then the individual molecules move, that is, the molecules increase in temperature (kinetic energy) or mix internally. For example, striking a surface with a hammer causes the surface to warm. Some objects are elastic and may store force as tension (potential energy); for example, tension in a spring (Fig. 1a). Tension forces are studied extensively in gating mechanosensitive ion channels^3^,^4^,^5^.

Surprisingly very little is known about kinetic components of force transduction in a biological membrane. As no system is perfectly elastic, a component of applied force must dissipate in the form of kinetic energy, raising the following questions: how much kinetic force is required to perturb a biological membrane and how does this energy affect the plasma membrane and mechanosensitive proteins?

Previous work showed that the plasma membrane is comprised of heterogeneous lipids that diffuse laterally and spontaneously partition into lipid rafts (Supplementary Fig. 1); also referred to as lipid microdomains^8^. Thermodynamically, we expect force to disrupt lipid partitioning through an increase in kinetic energy and overcome the entropic cost of demixing^9^. Since signalling lipids and mechanosensitive proteins often reside compartmentalized within or outside of lipid rafts^7^, we hypothesized that force-induced mixing of lipid compartments in a biological membrane could activate a mechanosensitive protein and transduce a biological signal. For example, if an enzyme resides in a raft and the enzyme's substrate resides outside of a raft, then mechanical disruption of the raft exposes the enzyme to its substrate producing a mechanically activated signal (see Fig. 1b).

Here we observe real time assembly and disassembly of rafts in live cells using fast super resolution imaging. We find the enzyme phospholipase D (PLD) localizes to lipid rafts and that mechanical force causes PLD to leave the lipid raft, interact with PIP_2_ microdomains and activate through substrate presentation. We conclude that lipid order and subsequent disruption is a membrane-delimited mechanosensor capable of activating a protein through an induced change in lipid localization.

## Results

### Live-cell imaging reveals lipid raft assembly and disruption

To test our lipid disruption hypothesis, we first characterized the abundance and size of cholesterol-rich lipid rafts in mouse myoblast cells (C2C12). C2C12 cells were fixed and labelled with a GM1 raft-specific Alexa 647 Cholera toxin-B (CTxB) and super resolution images were acquired using 3D stochastic optical reconstruction microscopy (3D-dSTORM) (refs 10, 11, 12) for a median localization precision of ∼26 nm in the *x*- and *y*-axis. CTxB is a multivalent toxin; in order to minimize artefacts from potential crosslinking of rafts, we fixed the cells, prior to CTxB labelling (see also methods). CTxB rafts (CTx-rafts) had an average diameter of 56.9±46.1 nm (s.d.) and distributed into two populations. The most abundant rafts measured 30–70 nm in diameter; a second, larger group contained diameters varying from 100 to 400 nm. In previous studies, measurements carefully calculated in sphingomyelin-containing domains from epithelial cells measured less than 20 nm (ref. 13); early measurements in fixed fibroblast cells ranged from 20 to 900 nm (ref. 14).

**Figure 1:**
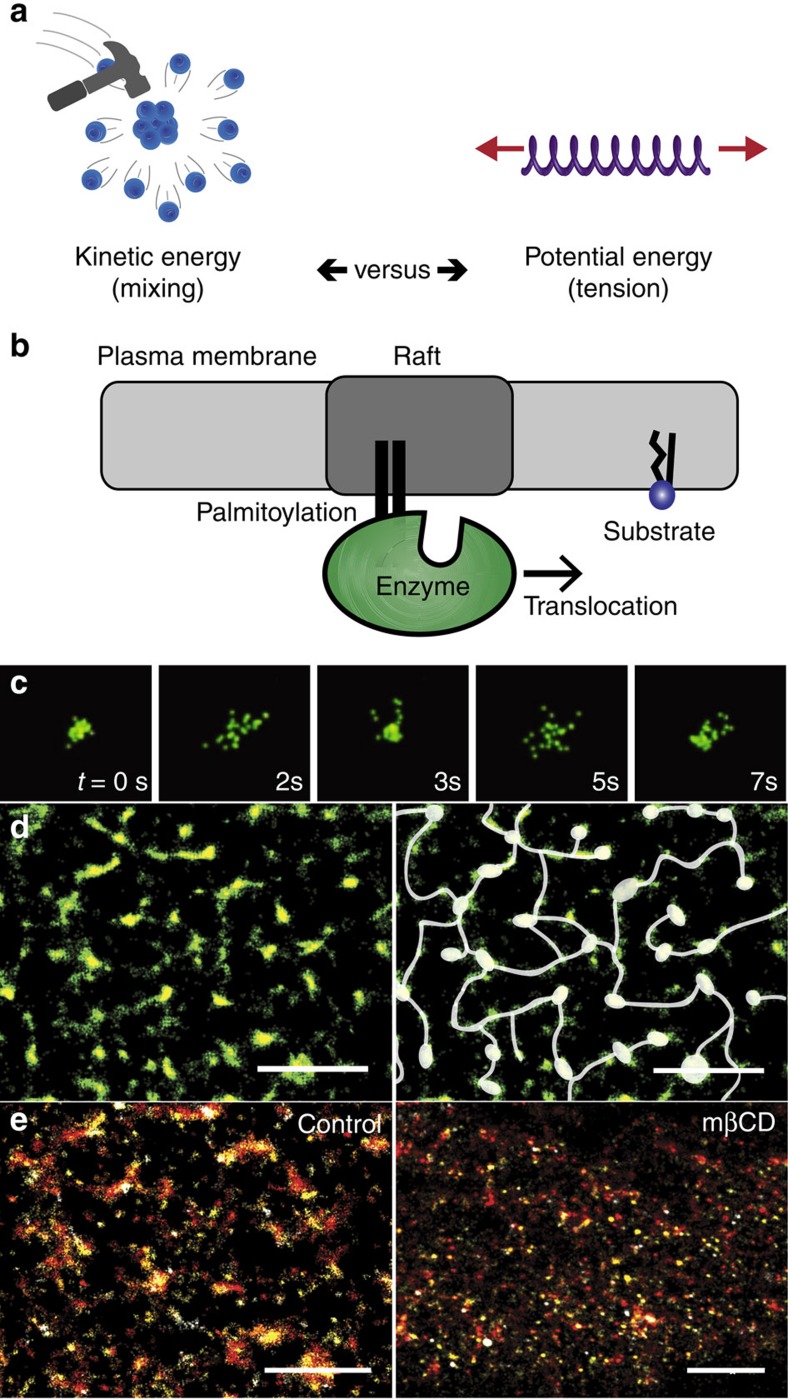
Live cell imaging of lipid raft disruption in C2C12 cells. (**a**) Diagram of the two major effects resulting from forces applied to a membrane. (**b**) Diagram of kinetic hypothesis for mechanical activation. An enzyme localized to a lipid raft is sequestered away from its substrate. Mechanically induced translocation of the enzyme from the raft leads to substrate access and enzyme activation. (**c**–**e**) dSTORM imaging of live C2C12 cells. (**c**) Single frames showing assembly and disassembly of a ∼125 nm CTx-raft (cropped from Supplementary movie S1). (**d**) Time averaged CTx-raft localization (movie S1); rafts dynamics are outlined with hubs and highways observed during live imaging (30 s), scale bar is 3 μm. The hubs are areas of high probability for large raft assembly and disassembly, while the highways allow for transient ordered trafficking of small particles between hubs (white tracing). (**e**) Time-dependent localization maps showing ordered domains were localized before (left), but rarely after mild mβCD treatment (100 μM) (right). Colours represent time; *t*=0 (dark red) to *t*=2.5 min (white); scale bar is 3 μm.

In order to understand the dynamics of these lipid rafts and ascertain their suitability as mechanosensors in the plasma membrane of C2C12 cells, we examined the dwell time and lateral movement of cholesterol rafts in real time using high-resolution 3D-dSTORM imaging on live cells. CTxB-labelled cells were monitored at 200 fps for a total of 2.5 min. We observed cholesterol rafts fluctuating between assembled and disassembled states (Fig. 1c, Supplementary Video 1) due to apparent thermal oscillations or vibration in the microscope. When averaged over time, the particles concentrated within localized regions of the membrane (Fig. 1d,e). Importantly, rafts imaged in live cells were comparable in size and distribution to rafts imaged in fixed cells, illustrating that potential CTxB crosslinking is minimal (Supplementary Fig. 2). The localization was consistent with lipid and protein diffusional barriers or ‘corrals' identified previously by single particle tracking^15^,^16^,^17^. However, the assembled and disassembled states were only revealed by our ability to monitor multiple particles simultaneously. Interestingly, in the disassembled state, the rafts diffused along preferred routes between two or more corrals (Fig. 1d). Lipids were known to move between corrals, but our ability to observe multiple particles simultaneously showed that this rapid diffusion occurred along defined routes. To better reflect this distinction we call these routes ‘highways' and the corrals ‘hubs'.

Recently, signalling lipids like phosphatidylinositol 4,5-bisphosphate (PIP_2_) have been found to exist in nanoscale regions of cells or lipid microdomains^18^,^19^. We investigated the distribution and size of PIP_2_ domains in C2C12 cells to compare their size and distribution to CTx-rafts. Cells were fixed, labelled with α-PIP_2_ antibodies and imaged with 3D-dSTORM with an observed median localization precision of 16 nm. PIP_2_ domains consisted of a single group of similarly sized rafts with an average diameter of 48.3±15.5 nm (s.d.), confirming previous analysis of their size^18^. PIP_2_ domains were not homogenously distributed across the cell but were present mainly on the distal ends of cells (Supplementary Fig. 3). Labelling artefacts from PIP_2_ antibodies are unlikely, as PIP_2_ is polyunsaturated and expected to reside in the liquid disordered region of the membrane^20^.

**Figure 2:**
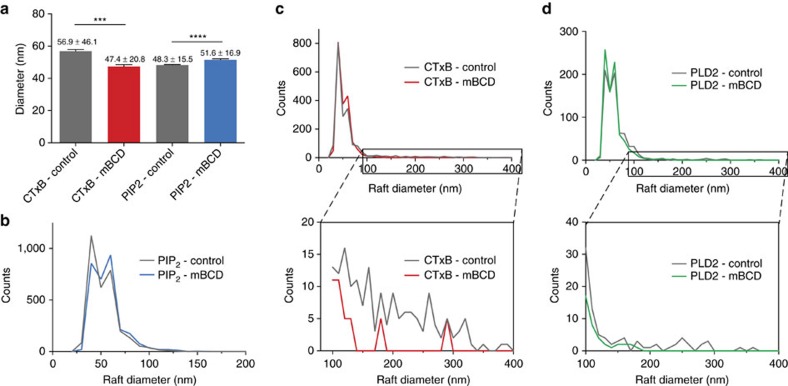
Quantitative effect of membrane disruption on raft diameter. (**a**) Lipid raft sizes were determined for CTxB and PIP_2_ domains before and after treatment with mβCD (reported as means±s.d.) ***P<0.001, ****P<0.0001 by two-tailed Student's *t*-test. A reduction of cholesterol by mβCD shows a decrease in the overall size of CTx-rafts and an increase in the average diameter of PIP_2_ domains. (**b**–**d**) Histograms of particle size distribution. (**b**) Analysis of PIP_2_ domains after mβCD treatment shows the size increase occurs as a result of a shift in small, well-defined, particles (<100 nm) to a slightly larger diameter. (**c**) In contrast, CTx-rafts show little change in particles <100 nm in diameter and an almost total loss of a heterogeneous population of rafts >100 nm in diameter (see c. zoom). (**d**) Disruption of PLD2 labelled rafts was similar to CTx-rafts with the change occurring mostly as a result of the elimination of rafts >100 nm (see d. zoom).

To test the effect of raft disruption on the organization of signalling lipids, we depleted cholesterol from the membrane of C2C12 cells. GM1 rafts require cholesterol for proper organization of the rafts^21^. Depletion of cholesterol by methyl-beta-cyclodextrin (mβCD) sequesters cholesterol from the cells, causing disruption of the raft^21^. Addition of low concentrations of mβCD (100 μM) was followed by fixation and labelling. We observed near-complete mixing of CTx-rafts and bulk lipids which was similarly observed in live-cell imaging (Fig. 1e, Supplementary Video 2). This correlated with an overall reduction in the size of CTx-rafts (Fig. 2a,c) and highlights the importance of cholesterol for corral formation in cholesterol rafts. PIP_2_ rafts showed little change in size or local distribution in response to mβCD (Fig. 2a,b), indirectly confirming that PIP_2_ domains are a separate class of microdomains which are cholesterol independent^18^.

### Cholesterol rafts control PLD2 localization and activity

Next we sought to understand how mechanical disruption of rafts affects the localization and activity of proteins in the plasma membrane. In order to eliminate confounding input from membrane tension, we selected a soluble enzyme, PLD, which lacks a transmembrane domain but localizes to the plasma membrane through post-translational palmitoylation^22^. PLD is a well-characterized lipase that hydrolyzes phosphatidylcholine (PC) to generate the short-lived signalling lipid phosphatidic acid (PA) (Supplementary Fig. 4). Importantly, PLD activation is an initial step in the transduction of force in muscle mechanosensation and cell growth^23^ (Supplementary Fig. 4).

We reasoned that the saturated palmitoyl groups of PLD (ref. 22) could localize PLD to cholesterol rich rafts^24^ away from its substrate PC (refs 20, 25). Furthermore, PIP_2_ activates PLD (ref. 26) and resides with PC outside of cholesterol rafts in distinct domains characterized above (see also Supplementary Fig. 1). This localization of PLD to a cholesterol raft, away from its substrate (PC) and activator (PIP_2_), suggests it could be activated by lipid mixing.

**Figure 3:**
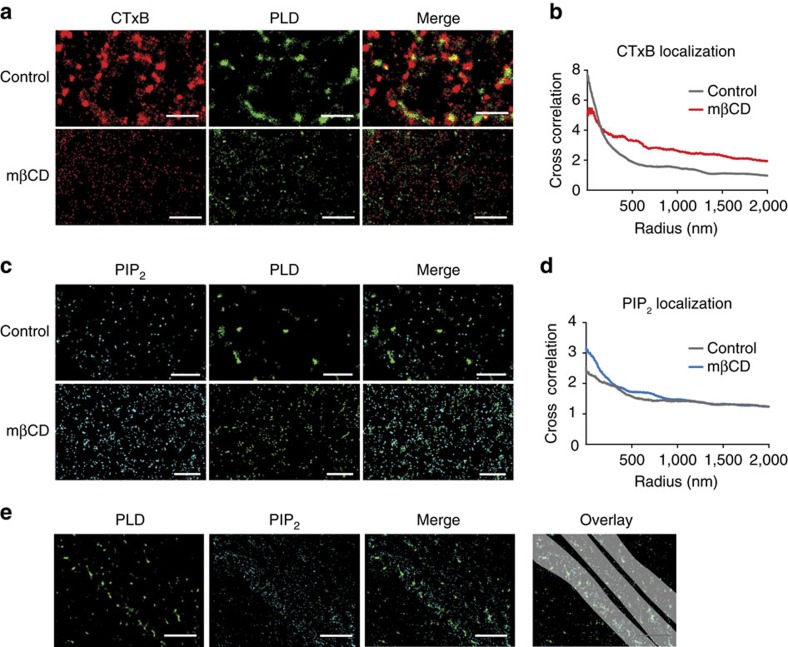
Raft disruption translocates PLD from CTx-rafts to PIP_2_ domains. (**a**) Fluorescently labelled PLD2 enzyme (green) colocalizes (merged control) with CTxB-labelled cholesterol rafts (red) before, but not after, treatment with mβCD confirmed by cross-correlation analysis in **b**. In contrast, prior to mβCD treatment PLD2 localizes away from PIP_2_ domains (cyan) (**c**), increasing in correlation only after treatment with mβCD (**d**). Bars in **a** and **c** represent 2 μm. (**e**) On the distal ends of cells, PIP_2_ domains and PLD were observed in striped, concentrated regions. An overlay of high-density regions of PIP_2_ and PLD2 domains highlights this observation. Bars in **e** represent 4 μm.

To confirm PLD localization to CTx-rafts, we labelled both GM1 rafts and endogenous PLD isoform2 (PLD2) and imaged the cells using 3D-dSTORM as described above with a resulting mean localization precision of 13.8 nm for PLD2 and 6.0 nm for CTx-rafts. (Fig. 3a). We observed a strong dependency on PLD2 localization to CTx-rafts. Since traditional colocalization calculations cannot be accurately used with dSTORM, a cross-correlation analysis^27^ was used, which quantifies the strength of association between two channels at a given distance. Analysis of PLD2 to CTx-rafts confirmed the expected association (Fig. 3b) allowing us to conclude that in a resting state, the majority of PLD2 is located within prototypic cholesterol rafts as predicted.

We then observed the effect of raft disruption on PLD localization. As expected, the localization of PLD2 to CTxB was dependent on raft integrity. After treatment with mβCD, cross-correlation analysis showed decreased association between PLD2 and CTxB when compared to control (Fig. 3b). Additionally, analysis of the PLD2 signal before and after mβCD treatment showed an ∼8 nm decrease in raft size due to a loss of rafts >100 nm in diameter, similar to the change in size of CTx-rafts (Fig. 2d). Treatment with latrunculin-A (lat-A) did not disrupt the localization of PLD2 to CTx-rafts (Supplementary Fig. 5b), indicating that the localization of PLD2 is not dependent on the cytoskeleton. These data suggest that the dwell time, localization and fluidity of CTx-rafts are suitable for their putative role in activation by raft disruption and that proteins localized to these rafts are subject to disruption by similar forces.

If cholesterol rafts (which contain PLD2) are separated from PIP_2_ domains (Fig. 3a), then we expect PLD2 is separated from PIP_2_ domains. As expected, direct imaging of PLD2 and PIP_2_ in the plasma membrane of C2C12 cells showed a clear separation of PLD2 from PIP_2_ (Fig. 3c). Interestingly, PLD2 localized cellularly with PIP_2_ to the same distal ends of the cell. Analysis of the mean distance between PLD and PIP_2_ molecules in these regions was 41 nm (Fig. 3e). After disruption of cholesterol rafts with mβCD, the strength of the local interaction between PLD2 and PIP_2_ increased (Fig. 3d), a result opposite of CTx-rafts with PLD2. This suggests PLD2 leaves a cholesterol raft and interacts with PIP_2_ domains.

### PLD2 activity increases in response to mechanical shear

**Figure 4:**
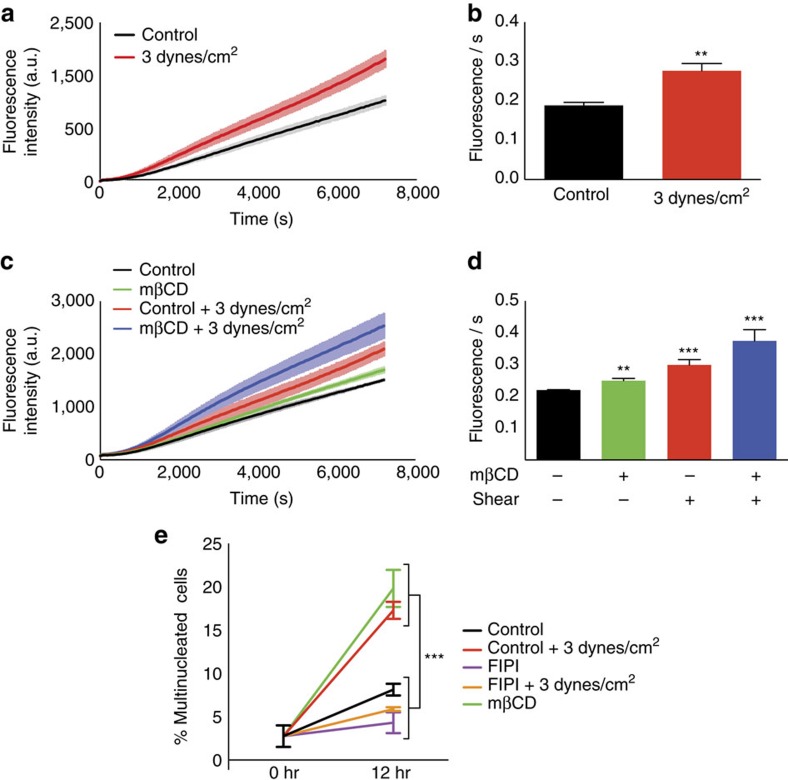
Mechanical disruption of lipid rafts activates PLD enzyme. (**a**) shows the effect of shear force on PLD activity in C2C12 cells. (**c**) shows an increase in PLD activity due to mβCD alone. (**b**) and (**d**) are a quantification of (**a**) and (**c**), (*n*=4–8, **P<0.01, ***P<0.001, Student's *t*-test). (**e**) PLD2 mediates the transduction of force to trigger cell differentiation. Mechanical force or mβCD treatment alone is able to increase the rate of differentiation, whereas the PLD2 antagonist FIPI inhibits this differentiation (*n*=4–5). Error bars are reported as mean±s.e.m.

To test direct activation of PLD2 in response to force we developed a live, fluorescent PLD assay. The assay monitors PLD product release in real time from intact cells. We applied shear force to C2C12 cells and monitored PLD2 activity. Three dynes cm^−2^ orbital shear force robustly activated PLD2 when applied in 20 s bursts every minute for the course of the assay (Supplementary Fig. 6 and Fig. 4a). To confirm this assay was specific for PLD activity, we blocked PLD activity by treating cells with 1 μM 5-Fluoro-2-indolyl des-chlorohalopemide (FIPI), a high affinity PLD antagonist (Supplementary Fig. 6a). Inhibition with the PLD-specific inhibitors VU0364739 and VU035959 during shear confirmed that PLD2 was the major contributor to this signal (Supplementary Fig. 6b). Muscle cells normally experience physiological forces of 1–3 dynes cm^−2^ (ref. 28). Our result suggests that PLD is directly activated by mechanical shear during normal biological activity.

To determine if PLD activation is due to lipid raft disruption, we chemically disrupted lipid rafts with mβCD. Similar to shear force, mβCD alone was able to increase PLD2 activation (Fig. 4c,d). We then combined shear with mβCD disruption and saw a synergistic increase in PLD2 activity (Fig. 4d). Since the increase was much greater than a mere additive effect, we conclude both mechanisms are acting along the same pathway, and raft disruption is the basis for the increased mechanical response. Treatment with lat-A showed no response (Supplementary Fig. 5) indicating that the mechanical response was not mediated by the cytoskeleton.

Shear force accelerates differentiation in muscle cells^29^. To determine if shear-induced differentiation can be replicated by raft disruption we monitored C2C12 cell differentiation during shear and mβCD treatments. Cells subjected to shear force differentiated more quickly than non-shear cells as monitored by the formation of multinucleated myotubes (Supplementary Fig. 7). This increased rate of differentiation was also observed during stimulation with mβCD alone and was inhibited by FIPI (Fig. 4e). This indicates that myocyte differentiation is mediated by the activation of PLD during mechanical raft disruption.

## Discussion

**Figure 5:**
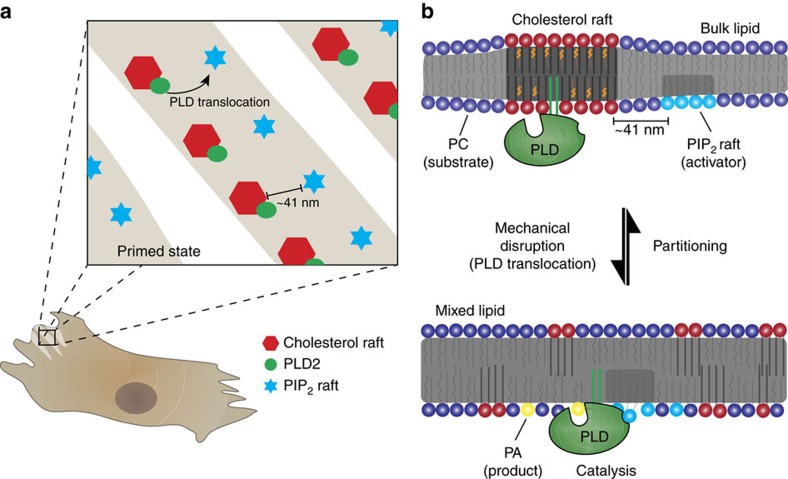
A kinetic (non-tension) mechanism for mechanosensation. CTxB- and PIP_2_ domains exist in tandem, often in localized regions at the distal ends of cells (**a**). The proximal separation of PLD and PIP_2_ allows for a ‘primed state', decreasing the latency between PLD2 translocation from cholesterol rafts to enzymatic activation by PIP_2_ (∼650 μs). (**b**) Mechanical force increases the kinetic energy in the membrane. The increase in kinetic energy overcomes the miscibility of the raft components, leading to raft disruption. During this disruption, PLD2 experiences increased access to PC and PIP_2_ resulting in the increased synthesis of the signalling lipid, PA.

We propose a kinetic model (non-tension) of force transduction (Fig. 5) in which disruption of lipid order results in transduction of force absent classical tension. Recent studies have developed mechanisms of mechanical transduction based on the elastic properties of the plasma membrane^3^,^4^,^5^. However, how a fluid plasma membrane may respond to lipid mixing during mechanical stimuli has not been investigated. Here, our data show that enzymes localized to lipid rafts, specifically PLD2, respond to lipid raft dispersion during a mechanical stimulus due to the increase in substrate accessibility.

The entropic cost of demixing^9^ drives the mechanosensitive process. Apart from activation by the input of energy (mechanical disruption), lowering the entropic cost of demixing allows a previously hindered process to become spontaneous. For example, the addition of mβCD induced mixing by lowering the entropic barrier and releasing the kinetic energy otherwise stored as entropy. Cells are known to exert homeostatic control over membrane composition through production of specific lipid species^30^. In our kinetic model, membrane composition, viscosity and raft size are used by the cell to attenuate force-induced lipid mixing, setting a baseline (that is, entropic cost of demixing) for the sensitivity of a membrane to force. We use the term ‘kinetic' to infer motion and to better differentiate our model from previous models proposed for tension.

Furthermore, our live imaging shows that a cell dynamically regulates the movement of lipids between rafts. This combined with a rafts ability to localize proteins and lipids ‘primes' the signalling pathways for mixing (Figs 3e and 5a). For example, the close proximity of PIP_2_ domains with PLD2 optimally positions the proteins to respond to rapid mechanical stimulation. We calculated the expected latency (response time) of lipid mixing after a mechanical stimulus based on the translocation of a single particle towards a stationary target (see methods). Our conservative estimate, based on known diffusion rates of lipids^31^ and the separation distances calculated from our PIP_2_ rafts and CTx-raft images, resulted in a latency of 650 μs, a threshold far below the activation latency of known mechanosensitive proteins^32^ and approaching the short latency predicted in auditory hair cells^33^. Our calculation does not account for diffuse PIP_2_ molecules, which would further decrease the latency. We expect increases in the concentration of signalling lipids or proteins will decrease the distance separating the components and lead to faster response times and may account for the high concentrations of PIP_2_ at the distal regions of auditory hair cells^34^.

Additionally, the rafts' ability to compartmentalize proteins and regulate their activity defines microdomains as a type of micro-organelle that initiates a rapid cellular pathway in response to external stimulus. Collectively, the cellular control over lipid rafts in the plasma membrane is likely to affect numerous mechanosensitive proteins as many mechanosensitive ion channels and enzymes are sensitive to raft formation and anionic signalling lipids^20^,^35^. PA has emerged as a broad signalling molecule in immune activation, wound healing, vesicular trafficking, secretion, endocytosis, cell survival and osmotic stress in plants among others^36^. Role of raft disruption will need to be investigated on important downstream targets such as mTOR and raf-1 (ref. 36).

Lastly, our mechanism of activation by substrate presentation through translocation to disordered lipids may explain why PLD tends to hydrolyse mono and polyunsaturated containing lipids^37^. The unsaturated lipids are more likely to partition with PIP_2_ in the disordered region of the membrane. When PLD is active near PIP_2_, the availability of substrate dictates the composition of lipid that is hydrolysed. In this manner the incorporation of a particular acyl chain into different types of other potential PLD substrates (for example, phosphatidlethanolamine or various amine containing plasmologen lipids) may dictate PLD substrate specificity. Other soluble enzymes with unexplained preference for acyl chain unsaturation will likely work through a similar mechanism of localization and substrate presentation. We expect the principles laid out here will contribute to a paradigm widely applicable in cell biology.

Taken together, our results outline a non-tension mechanism for the transduction of mechanical force in PLD signalling. Mechanical disruption of cholesterol rafts exposes PLD to the lipid activator PIP_2_ and lipid substrate (for example, PC) leading to a mechanically induced production of PA and downstream signalling (Fig. 5b).

## Methods

### hPLD2-GFP cDNA construct

MGC Human PLD2 cDNA (Accession: BC015033.1, cDNA clone MGC: 9152 Clone ID: 3907928) was purchased from Thermo Scientific. An upstream XhoI restriction enzyme digestion site and a downstream EcoRI site were introduced into the hPLD2 gene by PCR. The primers used were as follows: hPLD2 forward, 5′-ACCCTCGAGCTA TGACGGCGACCCCTG-3′; hPLD2 reverse, 5′-GCGAATTCCTATGTCCACACTTCTAG G-3′. The PCR fragment was cloned into the pcDNA3 vector with chitin binding domain (CBD), GFP tag, and prescission protease domain (PPX) and verified by sequencing. The final construct coded for an N-terminal GFP tagged hPLD2 with chitin binding site (CBD-GFP-PPX-hPLD2).

### Cell culture

All cells were grown in Dulbecco's modified Eagle medium (DMEM) containing 10% fetal bovine serum (FBS) and 1% penicillin/streptomycin unless otherwise noted. C2C12 cells were changed to a serum-free media containing no FBS or antibiotics 24 h prior to experimentation unless otherwise noted. For the *in vivo* assay, phosphate-buffered saline (PBS)-glucose buffer contained D-glucose (20 mM) in PBS (VWR, 45000-446).

### Fixed cell preparation

C2C12 cells were grown to 80% confluence and then allowed to differentiate overnight in serum-free media. Cells were rinsed, treated as needed, and then fixed with 3% paraformaldehyde and 0.1% glutaraldehyde for 10 min to fix both protein and lipids. Glutaraldehyde was reduced with 0.1% NaBH4 for 7 min followed by three 10 min washes with PBS. Cells were permeabilized for 15 min with 0.2% Triton X-100 and blocked with 10% bovine serum albumin (BSA)/0.05% Triton/PBS at room temperature (rt) for 90 min. Primary antibody was added in a solution of 5% BSA/0.05% Triton/PBS for 60 min at rt followed by five washes with 1%BSA/0.05% Triton/PBS for 15 min each. Secondary antibody was added in the same buffer as primary for 30 min at rt followed by five washes as above. A single 5 min wash with PBS was followed by a post-fix with fixing mixture, as above, for 10 min w/o shaking. This was followed by three 5 min washes with PBS and two 3 min washes with dH_2_O. Cells only receiving CTxB treatment were not permeabilized.

### Super-resolution 3D-dSTORM imaging

Images were recorded with a Vutara 352 super-resolution microscope (Bruker Nano Surfaces, Salt Lake City, UT, USA) which is based on the 3D Biplane approach^38^. Super-resolution images were captured using a Hamamatsu ORCA Flash4.0 sCMOS camera and a × 60 water objective with numerical aperture 1.2. Data were analysed by the Vutara SRX software (version 5.21.13). Single molecules were identified by their brightness frame by frame after removing the background. Identified particles were then localized in three dimensions by fitting the raw data in a customizable region of interest (typically 16 × 16 pixels) centred on each particle in each plane with a 3D model function that was obtained from recorded bead data sets. Fit results were stored as data lists for further analysis.

Fixed samples were imaged using a 647 nm and 561 nm excitation lasers, respectively, and 405 nm activation laser in photoswitching buffer comprising of 20 mM cysteamine, 1% betamercaptoethanol and oxygen scavengers (glucose oxidase and catalase) in 50 mM Tris+10 mM buffer +10% glucose at pH 8.0 at 50 Hz and maximal powers of 647 nm, 561 nm and 405 lasers set to 8, 10 and 0.05 kW cm^−2^ respectively. Live cell imaging was performed in DMEM supplemented with oxygen scavengers and 0.1% betamercaptoethanol in 50 mM Tris+10 mM buffer +2% glucose. An autocorrelative algorithm^38^ was used to correct for drift correction.

Pair correlation and cluster analysis was performed using the Statistical Analysis package in the Vutara SRX software^39^,^40^,^41^,^42^,^43^. Briefly, the cross-correlation function, *c*(*r*), quantifies the increased probability of finding signal a distance *r* away from a signal of a different channel^27^. Cluster size was determined by measuring the full width half max (FWHM) circumference for clusters with >5 particles.

### Super resolution SIM microscopy

Structured Illumination Microscopy (SIM) was performed on a Zeiss ELYRA PS.1 microscope with a × 63/1.4NA objective and recorded using an Andor iXon 885 EMCCD (1024 × 1024 pixels, 8 × 8 μm pixel size, 65% QE), for a maximum field of view of 80 × 80 μm. Raw SIM data sets were acquired by projecting grids onto the sample generated from the interference of the 0th and ±1st diffraction orders from a phase grating. For the 405, 488, 561 and 642 nm excitation, phase gratings of spacing 23, 28, 34 and 34 μm (respectively) were used to generate illumination grids for maximum resolution improvement of each colour. Each super-resolved image required five grid shifts (phases) and three grid rotations for a total of 15 images per super-resolved z-plane per colour. The ELYRA PS.1 system's maximum laser output was 50, 200, 200, 150 mW (respectively), with a dedicated ND filter wheel for each laser for fine power control. During acquisition, laser power, camera exposure time and camera gain were adjusted so that high contrast images (50% camera dynamic range, 16 bit) were acquired. For most images, a camera exposure time of 50 ms was used. For 3D images, z-stacks were acquired using a z-piezo stage insert by PI (PI-737). Images were reconstructed through a proprietary Zeiss Fourier-based algorithm. FRET images were obtained by excitation at 488 nm with the detector set to collect wavelengths from 565 nm and above. Intensity of excitation was set to maximize FRET intensity for each image. JACoP 2.0 addin for ImageJ was used to calculate the colocalization on the area of interest.

### Zeiss airyscan microscopy

Airyscan microscopy was performed on a Zeiss 880, AxioObserver with a Plan-Apochromat × 63/1.4 DIC M27 objective and recorded using an Airyscan detector in superresolution mode. During acquisition, laser power and detector exposure time were adjusted so that high contrast images were acquired. Raw Airyscan images were processed by the Zeiss ZEN software using automatic settings. Buffers for control image consisted of DMEM w/o phenol red supplemented with 2% glucose.

### PLD *in vivo* assay

hPLD2 transfected HEK cells (HEK-hPLD2, >5 × 10^4^ cells per well to ensure confluence) and C2C12 cells were seeded into 96-well culture plates. HEK-hPLD2 cells were incubated at 37 °C for 3 h in DMEM media until the cells were fully attached to the plate and C2C12 cells were switched to serum-free media as described above. The attached cells were washed and incubated with 50 μl of PBS-glucose or with PBS-glucose buffer containing treatment. The PLD assay reactions were begun by quickly adding 50 μl of working solution containing 100 μM Amplex red, 2 U per ml horseradish peroxidase, 0.2 U per ml choline oxidase and 60 μM dioctanoyl phosphatidylcholine (C8PC) in PBS-glucose buffer. Final concentration of each component was twofold lower in the final reaction volume. Fluorescence was measured with a fluorescence microplate reader (Tecan Infinite 200 PRO) at 37 °C for 2 h with Ex/Em of 530/585 nm. Each data point and background was measured in triplicate. The background (reaction mixture lacking cells) was subtracted from each sample. Samples were normalized to the control and were then graphed and statistically analysed with GraphPad Prism software (v6.0f).

For the mechanical agitation assay in HEK cells, we introduced a crude mechanical stimulation to the plasma membrane of HEK-hPLD2 cells. Cells were trypsin treated, counted, washed with PBS, and then re-suspended in PBS-glucose buffer and mechanically activated by gently pipetting up and down (15 × , two cycles with 3 min rest). Cells were distributed at 5 × 10^4^ cells per micro well (50 μl cell suspension solution) and monitored for viability (> 95%). The mechanical stimulation step required 7–10 min followed by immediate assaying of PLD activity as described for resting cells above.

### Mechanical (shear) and chemical raft disruption

For applied mechanical force, C2C12 cells were plated at 10K cells per well in a 96-well, clear bottomed plate and allowed to adhere overnight. Cells were partially differentiated as explained above. On the following day, assay reagents were added and cells were placed in a microplate reader at 37 °C. The plate reader was programmed to agitate the cells using orbital rotations from 1 to 6 mm for 20 s every minute for the duration of the assay. Readings were taken every minute for each well and data analysed as described above. Shear force was calculated using the orbital shear equation, 
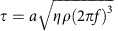
, where *a* is the orbital radius, *ρ* is the density of the culture medium, *η* is the viscosity and *f* is the frequency of rotation (rotation/s) as described previously^44^.

For cell differentiation assays cells were grown to >90% confluency and allowed to partially differentiate in 3% FBS-DMEM for 12 h. Cells were then subjected to the treatments noted every 3 h for a total of five treatments. Cells were immediately imaged to determine rate of differentiation between treatments.

For chemical disruption of lipid rafts, HEK and C2C12 cells were prepared and the following compounds were added to the reaction buffer at the noted concentrations: rapamycin (150 nM) (LC Laboratories, R-5000); C8-PA (Avanti, 830842P) (30 μM); mβCD (300–1,000 μM) (Fisher, AC37711-0050); FIPI (Millipore, 528245); latrunculin A (latA) (Cayman Chemicals, 10010630). PLD specificity was confirmed by FIPI inhibition for all conditions.

### Particle calculation

Latency of particle interaction was calculated using the mean-square displacement equation for Brownian or random walk motion:


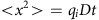


where <*x*^2^> is the displacement distance, *q*_*i*_ is a dimensionality constant and equals 4 for 2 dimensions, *D* is the diffusion coefficient which was set at 15 μm^2^ s^−1^, and *t* is time in seconds. <*x*^2^> was determined using the median value of 41 nm found by performing the nearest neighbour analysis between identified PLD2 and PIP_2_ particles within the Vutara SRX application.

Raft distances were determined by first identifying rafts and determining the distance between their centres using the nearest neighbour analysis in the Vutara SRX application. Bimodal distributions (where applicable) were deconvoluted using the normalmixEM portion of the mixtools package in R and the means of the individual Gaussian distributions used as the appropriate distances.

### Reagents and statistics

10-Acetyl-3,7-dihydroxyphenoxazine (Amplex red)(Cayman Chemicals); Choline oxidase (MP Biomedicals); horseradish peroxidase; mβCD (Acros organics); 1,2-dioctanoyl-sn-glycero-3-phosphocoline (C8-PC) (Avanti Polar Lipids Inc.). Amplex red was first dissolved in dimethyl sulfoxide to prepare 10 mM stock solutions and stored frozen at −20 °C, protected from light for up to 6 months. C2C12 cells (ATCC CRL-1772). Corning Cellgro DMEM (Fisher Scientific, MT10013CV). Cellgrow FBS (VWR, SH3039603). Goat α-rabbit IgG (VWR PI-1000) 1:2,000; α-PLD2 (E1Y9L, Cell Signaling #13891), 1:150; α-PtdIns(4,5)P2 (Echelon, Z-P045), 1:200. Donkey α-mouse Cy3B antibodies were a gift from Manasa Gudheti. PLD1 (VU0359595) and PLD2 (VU0364739) inhibitors (500 nM) were a gift from Alex Brown.

Cluster analysis was performed using VutaraSRX cluster analysis tool over multiple images. GraphPad Prism was used to determine significance for raft size; CTxB (*n*=1,907), CTxB+mβCD (*n*=344), PIP_2_ control (*n*=3,076), PIP_2_+mβCD (*n*=778). Cell differentiation assay was performed single blind on images taken from multiple dishes (*n*=4 for each condition). All numbers are reported as mean±s.e.m. unless otherwise noted. As all samples were found to have a normal distribution, Student's *t*-test was used to determine significance with resultant *P*-values as reported.

### Data availability

The data that support the findings of this study are available from the corresponding author on reasonable request.

## Additional information

**How to cite this article:** Petersen, E. N. *et al*. Kinetic disruption of lipid rafts is a mechanosensor for phospholipase D. *Nat. Commun.* **7,** 13873 doi: 10.1038/ncomms13873 (2016).

**Publisher's note**: Springer Nature remains neutral with regard to jurisdictional claims in published maps and institutional affiliations.

## Supplementary Material

Supplementary Information Supplementary Figures 1-7

Supplementary Movie 1 Live cell imaging showing raft lateral mobility and dynamics. C2C12 cells were imaged using STORM at a rate of 200fps resulting in an average of 12 hits per frame. Each frame represents the overlap of 3, 0.25 s time frames, for a total of 0.75 s/frame. The video shows GM1-labeled rafts are localized and dynamic; rafts repeatedly formed and disassembled in unison. (see also Fig. 1c, e).

Supplementary Movie 2 Live cell imaging with mβCD treatment. C2C12 cell were imaged after treatment with 100μM mβCD and condensed as with Supplemental Movie 1. After a reduction in cholesterol, the large rafts are more dissembled and randomly dispersed across the membrane (see also Fig. 1e).
