# Supplementary material for: Kinetic disruption of lipid rafts is a mechanosensor for phospholipase D
Source: Nat Commun. 2016 Dec 15;7:13873. doi: 10.1038/ncomms13873 (PMC5171650; doi:10.1038/ncomms13873)
Supplement: Supplementary Information — Supplementary Figures 1-7 [file ncomms13873-s1.pdf]

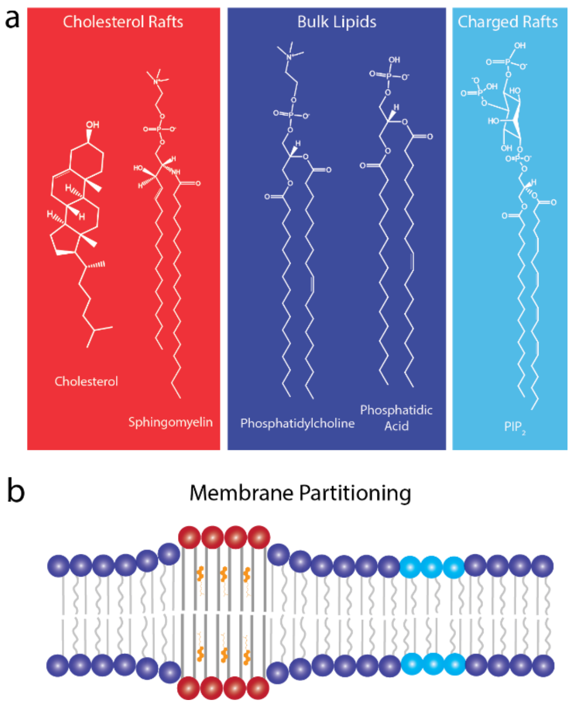


**Supplementary Fig. 1.**

Lipid composition and morphology of the cell membrane. (**a**) The plasma membrane is comprised of a heterogonous mixture of saturated, unsaturated, and charged lipids. Lipids spontaneously partition based on hydrophobicity and charge into microdomains or lipid rafts. (**b**) Cholesterol drives partitioning, thickening, and rigidity of saturated lipids, e.g. sphingomyelin by increasing favorable packing. Similarly, the anionic lipid $PIP_2$ clusters, driven by positive charges[17-18].

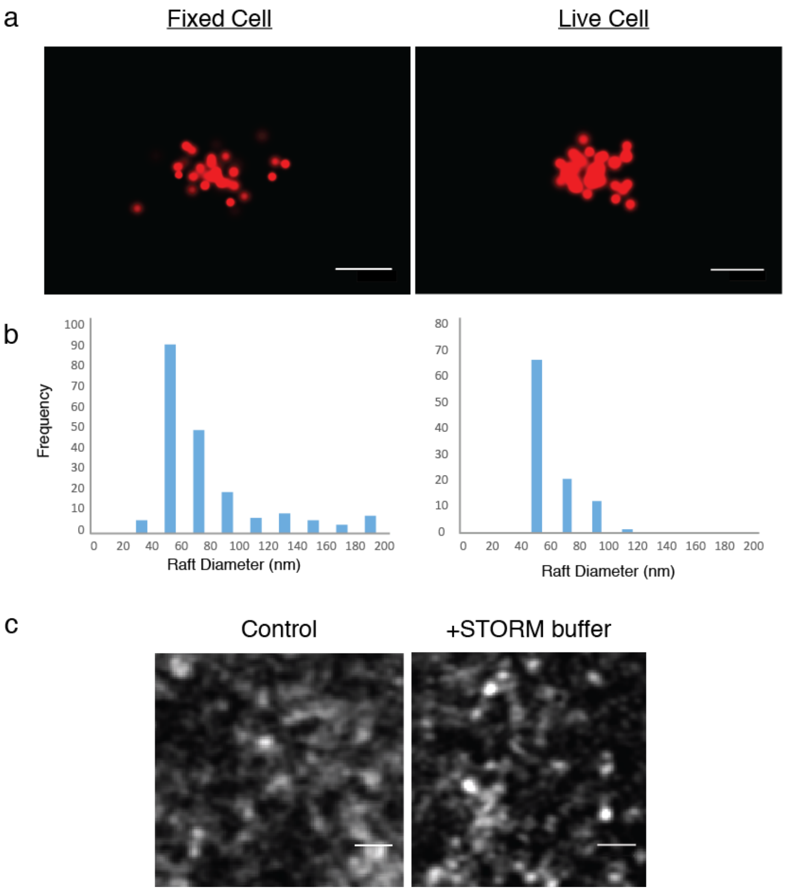


**Supplementary Fig. 2.**
Characterization of rafts in live and fixed cells. **(a)** Single representative rafts from both fixed and live imaging show similar sizes and distribution of individual particles after controlling for sampling density. Scale bar is 100nm. **(b)** Raft size distribution was analyzed to determine the effect of the sampling density on rafts identification and characterization. The average size of rafts was found to be relatively constant between fixed cells and live cell rafts with only a small percentage of large rafts being identified in fixed, but not live, cells. **(c)** To rule out potential artifacts from buffers and chemicals necessary for dSTORM imaging, we applied the media to the cells and imaged them using Airyscan super resolution imaging. Airyscan imaging shows that treatment of live C2C12 cells with STORM buffer does not have an effect on the size or distribution of rafts labeled with CTxB. Scale bar is 1µm.

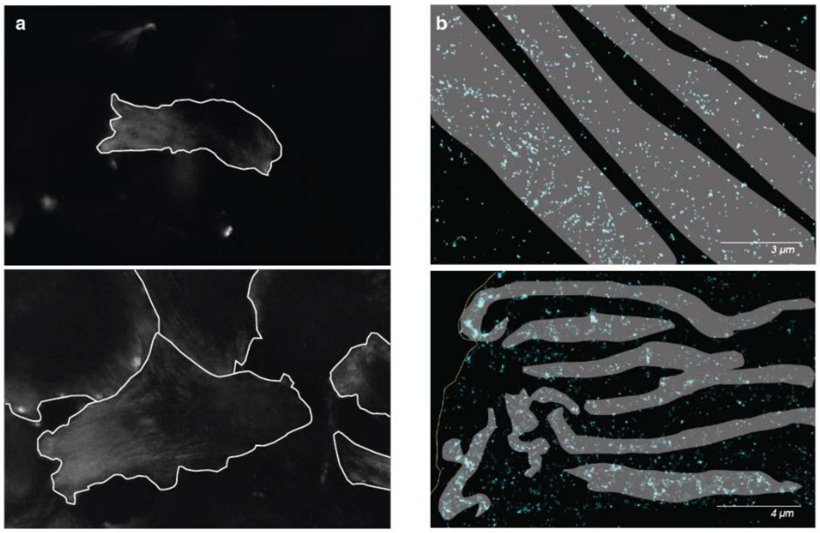


**Supplementary Fig. 3.**

Images of the $PIP_2$ organization seen in C2C12 cells. (**a**) Widefield images of $PIP_2$ labeled C2C12 cells with borders of in-focus cells added in white. Labeling shows $PIP_2$ is localized to the distal ends of the cells. $PIP_2$ is striped throughout the cell, indicating that there could be intracellular control of $PIP_2$ localization. Scale bar is 3 µm. (**b**) STORM imaging showing the organization of $PIP_2$ in high resolution. $PIP_2$ is often found in higher densities along linear paths on the distal ends of cells. Transparent white overlay has been added to emphasize the high-density regions. Scale bar is 4 µm.

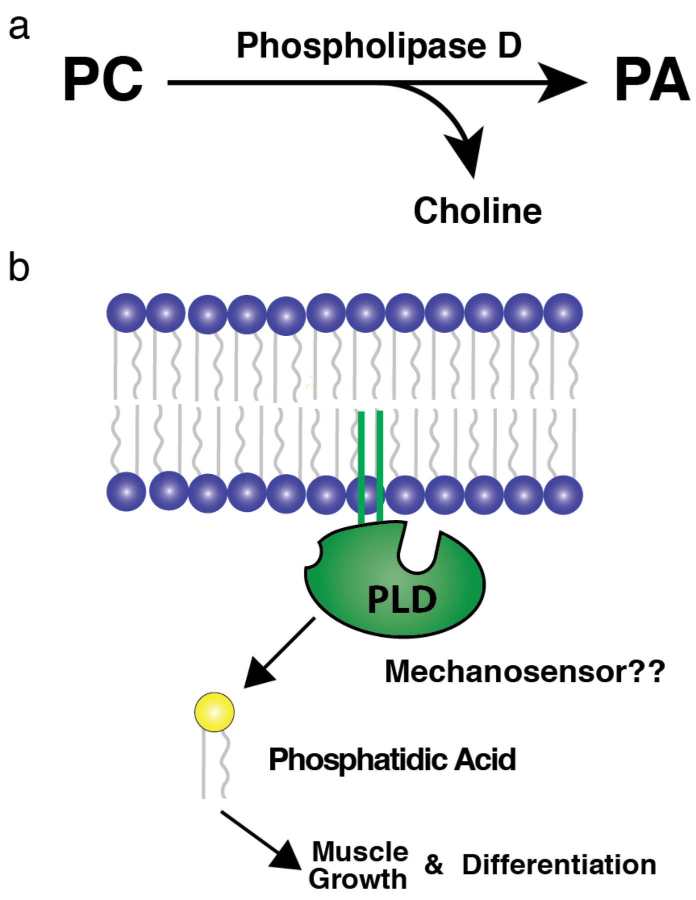


**Supplementary Fig. 4.**

Diagram of the PLD pathway in muscle cells. (**a**) PLD is an enzyme which catalyzes the hydrolysis of PC to PA with a loss of choline. (**b**) PLD is known to be among the first enzymes in the muscle mechanosensation pathway, although a definitive mechanosensor has not been identified. PLD activation (typically by a GPCR) leads to PA production, resulting in the activation of pathways involved in the growth and differentiation of muscle cells.

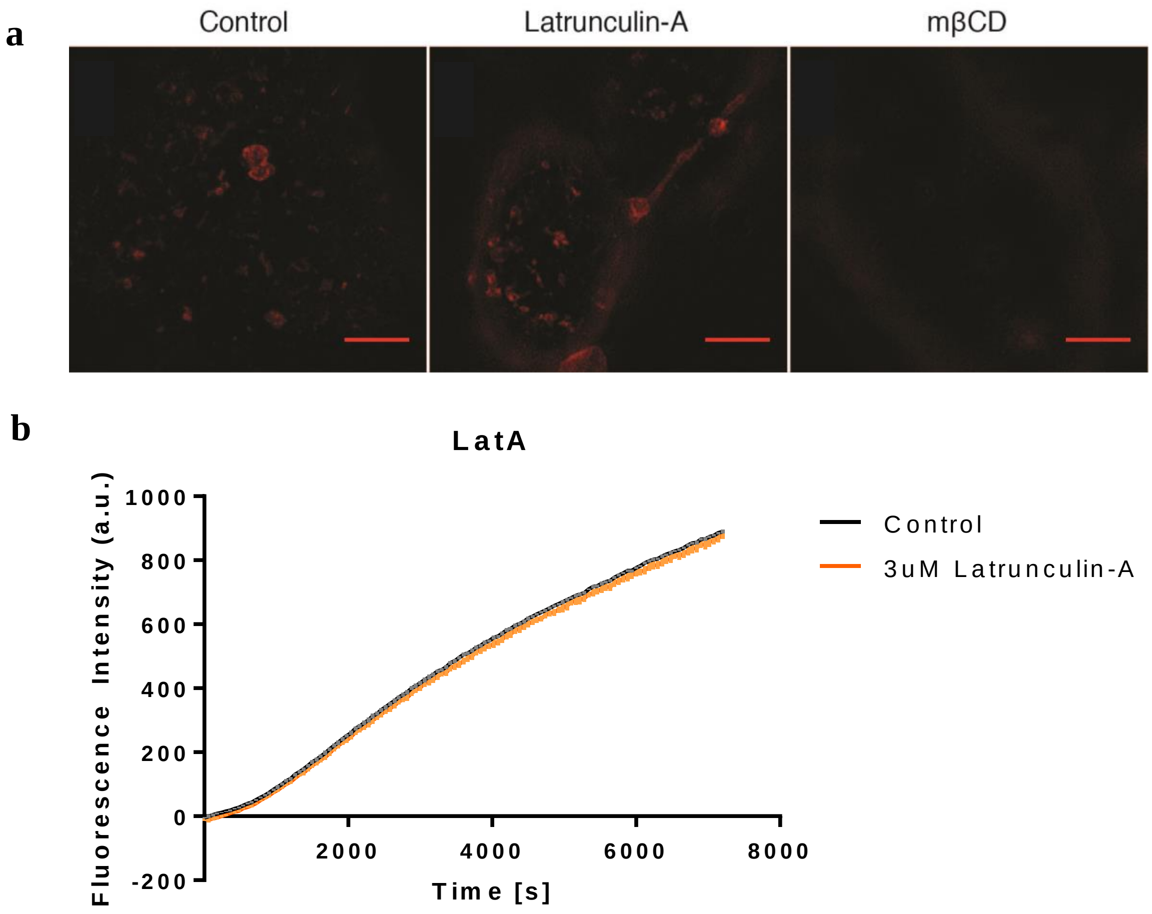


**Supplementary Fig 5.**
Role of the cytoskeleton in raft stabilization. (**a**) Förster resonance energy transfer (FRET) between PLD2::GFP and CTxB-Alexa 555 in HEK cells show the proximity of PLD2 to lipid rafts. This proximity is not changed after treatment with latrunculin-A but is lost after treatment with mβCD. Scale bar is 5 µm. (**b**) PLD activity is not affected by treatment with lat-A. Error is reported as mean ± s.e.m.

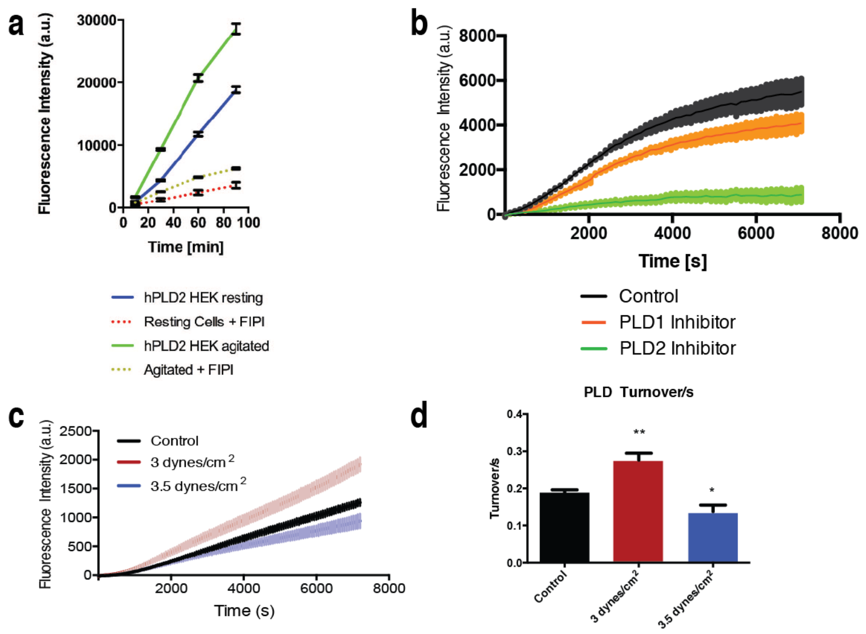


**Supplementary Fig. 6.**

PLD *in vivo* assay. (**a**) hPLD2 was overexpressed in HEK cells and the activity of PLD2 was quantified. Agitation of the cells increased PLD2 activity, while FIPI was able to inhibit PLD signal to background levels. (**b**) As expected, the majority of mechanically activated PLD is PLD2 (n=4). (**c-d**) The intensity of shear determines PLD activity. Application of 3 dynes/cm$^2$ lead to an increase of PLD activity ($p$ = 0.0056, $n$ = 6). Surprisingly, as little as 0.5 dynes/cm$^2$ above this level leads to the inactivation of PLD2 ($p$ = 0.036, $n$ = 4). 3.5 dynes/cm$^2$ is above the threshold for what muscle cells experience *in vivo* so the reduced activity may indicate a stress response. Error bars are mean ± s.e.m, Student's t-test.

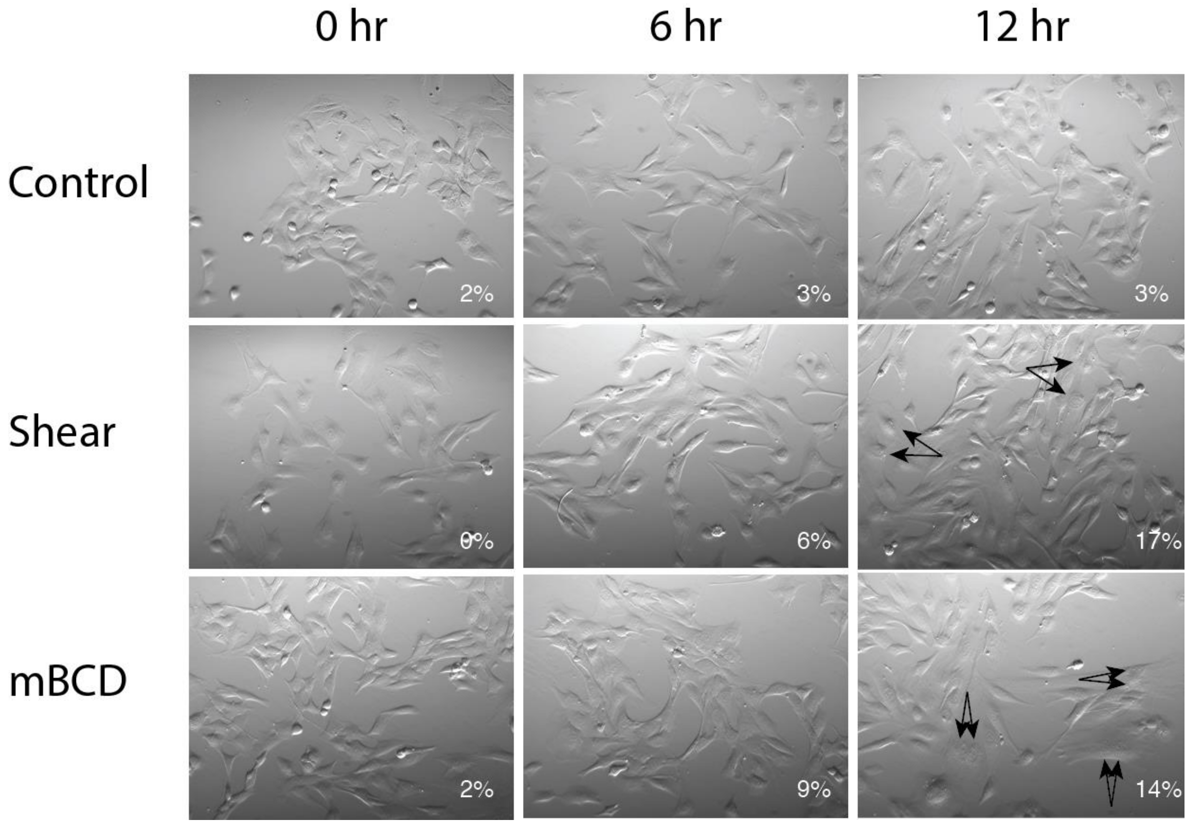


**Supplementary Fig. 7.**

Myocyte differentiation is mediated through the mechanical disruption of lipid rafts. Myocytes were allowed to differentiate under low-serum conditions with treatments being administered every 3 hours. All cells began under the same conditions. Cells were monitored for 12 hours and the number of nuclei involved in multinucleated cells (black arrows) were compared to the total number of nuclei imaged. Percentages of nuclei involved in multi-nucleated cells are shown in the bottom corner of each image.
